# Intercostal thickening fraction adds no value to diaphragm thickening fraction in healthy subjects undergoing noninvasive ventilation

**DOI:** 10.1038/s41598-026-40192-4

**Published:** 2026-02-17

**Authors:** Clara Hoermann, Luisa Sophie Drotleff, Burcu Link, Lena Doerflinger, Benjamin Neetz, Julia D. Michels-Zetsche, Felix J. F. Herth, Markward Britsch, Daniel Duerschmied, Simone Britsch, Simon Lindner

**Affiliations:** 1 Cardiology, Angiology, Haemostaseology, and Medical Intensive Care, Medical Centre Mannheim, Medical Faculty Mannheim, Heidelberg University, Mannheim, Germany; 2 European Centre for Angioscience (ECAS), Medical Faculty Mannheim, Medical Centre Mannheim and Medical Faculty Mannheim, German Centre for Cardiovascular Research (DZHK) Partner Site Heidelberg/Mannheim, and Centre for Cardiovascular Acute Medicine Mannheim (ZKAM), Heidelberg University, Mannheim, Germany; 3 Department of Pneumology and Critical Care, Thoraxklinik Heidelberg, Translational Lung Research Centre Heidelberg (TLRC-H), German Centre for Lung Research (DZL), University of Heidelberg, Heidelberg, Germany; 4 Department of Radiation Oncology, Medical Centre Mannheim, Medical Faculty Mannheim, Heidelberg University, Mannheim, Germany; 5 Medical Centre Mannheim, Medical Faculty Mannheim, DKFZ Hector Cancer Institute, Heidelberg University, Mannheim, Germany; 6 Mannheim Institute for Intelligent Systems in Medicine (MIISM), Heidelberg University, Heidelberg, Germany; 7 HMS Analytical Software GmbH, Heidelberg, Germany

**Keywords:** Respiratory muscles, ultrasonography, Noninvasive ventilation, Diseases, Health care, Medical research, Physiology

## Abstract

Assessing respiratory effort is essential for optimizing ventilatory support. While diaphragm thickening fraction (DTF) has been proposed as a bedside parameter, the role of intercostal muscle thickening fraction (ITF) remains unclear, especially during noninvasive ventilation. This study investigates the relationship between DTF, ITF and tidal oesophageal pressure swing (ΔP_oes_) as surrogate parameters for respiratory effort under different exercise loads. We conducted a physiological study in healthy volunteers with no contraindications to noninvasive ventilation. Participants completed three exercise sets on a semi-recumbent cycle ergometer. Each set consisted of five randomised phases with different ventilatory settings. ΔP_oes_ was measured using a nasogastric balloon catheter. Diaphragmatic and parasternal intercostal muscle thickening fractions (DTF and ITF) were assessed by ultrasound in B-mode. Repeated measures correlation and Friedman’s test were used to analyse associations and differences between parameters. 38 individuals were included in the study. Repeated measures correlation analysis revealed a moderate correlation between DTF and ΔP_oes_ (ρ = 0.419, *p* < 0.001), whereas ITF did not correlate with either parameter. Both DTF and ΔP_oes_ increased with exercise load and allowed discrimination between different exercise loads, although ΔP_oes_ provided a more accurate separation. In contrast, ITF did not discriminate between exercise phases. These findings suggest that DTF could be a useful indicator of respiratory effort during noninvasive ventilation. ITF may only be informative in the presence of diaphragmatic dysfunction. Further investigations in clinical populations are needed to test the hypotheses derived from this study.

**Supplementary Information:**

The online version contains supplementary material available at 10.1038/s41598-026-40192-4.

## Introduction

Noninvasive ventilation (NIV) is widely used in patients with acute respiratory failure to avoid invasive mechanical ventilation. By providing inspiratory pressure support (ΔP_insp_), NIV can both enhance oxygen delivery and compensate for the patient’s increased respiratory effort^1^. An assessment of respiratory effort is clinically relevant because increased respiratory effort seems to be a key factor in the development of acute respiratory failure and allows for the precise prediction of NIV success^2^, as both insufficient support and excessive pressures can have negative consequences. Excessive inspiratory pressures have been demonstrated to result in lung injury and diffuse alveolar damage^3^. Furthermore, high ΔP_insp_ have been shown to cause air leaks at the patient-mask interface, which in turn have been demonstrated to negatively affect patient-ventilator interaction. Dys- or asynchronies between the patient and the ventilator, such as ineffective triggering, can cause patient discomfort and can lead to NIV failure^4^. Therefore, estimating respiratory effort can help to individualise ΔP_insp_ settings, as optimal therapy should aim to maintain respiratory effort close to the level of resting spontaneous breathing^5^.

While the degree of ventilator support can usually be read from the inspiratory-expiratory pressure difference, it remains a challenge to assess the patient’s remaining effort and determine their actual need for support^6^. It has been established that the tidal oesophageal pressure swing (ΔP_oes_) is a reliable surrogate parameter for respiratory effort^7,8^. Preliminary studies have indicated that the diaphragm thickening fraction (DTF), determined by ultrasound, may also be a valuable parameter in this regard^9^. While the diaphragm is the primary respiratory muscle at rest^3^, accessory respiratory muscles become increasingly important as respiratory effort increases^10^. Inspiratory thickening of accessory respiratory muscles, such as the parasternal intercostals, might thus be a valuable addition to diaphragm ultrasound in cases of high respiratory effort^11^. Ultrasonographic measurement of in- and expiratory thickness of the parasternal intercostals has been described as a reliable, reproducible, and feasible bedside method^11^. Although several groups have proposed using ultrasound to assess ITF as an indicator of respiratory effort, the available evidence remains inconsistent across different study populations and experimental settings^12–15^.

However, to our knowledge, this noninvasive approach has not yet been evaluated during NIV, neither in the context of de-novo acute respiratory failure nor in patients receiving NIV after extubation. This differentiation is clinically relevant, as patients undergoing weaning from invasive mechanical ventilation often exhibit diaphragmatic atrophy or dysfunction due to prolonged mechanical ventilation^16^, whereas patients with de novo acute respiratory failure are more likely to have intact diaphragmatic and intercostal muscle function. As our study focused on healthy volunteers, we assumed that their respiratory muscle function was intact.

The primary aim of this study was to examine whether ITF provides superior discrimination between different levels of respiratory effort in healthy subjects undergoing NIV, compared to DTF. The secondary aim was to confirm our previous findings on the correlation of DTF and ΔP_oes_ in a randomized and blinded experimental setting.

## Methods

### Study design

This is a physiological study in healthy volunteers and was not prospectively registered.

### Outcome measures

Outcome measures for this study were the correlation of ITF, DTF and ΔP_oes_, as well as the ability of those index tests to differentiate between different exercise loads.

### Subjects

Healthy individuals aged 18 years or older, capable of providing written informed consent, were recruited for this study. Individuals were excluded from participation if they had a history of bleeding disorders, recurrent bleeding, or current anticoagulant therapy; if they had allergies to ultrasound gel or external materials containing plastic, such as NIV masks or gastric tubes; or if they refused to participate or had medical contraindications to NIV. The participants were seated on a semi-recumbent bicycle ergometer with a 45-degree torso incline.

### Patient and public involvement

Patients or the public were not involved in the design, or conduct, or reporting, or dissemination plans of our research.

### Workload determination

Exercise intensity was controlled using a cycle ergometer, allowing independent adjustment of workload and pedalling cadence. Effort perception was assessed using the Borg rating of perceived exertion (RPE) scale. A score of 10 on the Borg RPE scale corresponds to a light effort, while 15 indicates a demanding exertion^17^. Prior to the experiment, individual target workloads were determined in every participant by gradually increasing the wattage at 40 rpm until the predefined Borg RPE levels of 10 and 15 were reached. The corresponding wattages were then used as exercise intensities during the experimental phases.

### Measurement of oesophageal pressure swing

A nasogastric single-balloon PesoCath© oesophageal catheter (Loewenstein medical, Bad Ems Germany) was used. Insertion was facilitated by local anaesthesia of the nose and pharynx using lidocaine spray and catheter preparation with lidocaine gel. The probe was inserted via a nostril aiming at initial infra-diaphragmatic placement. The oesophageal balloon was then inflated with 6 ml of air, followed by deflation of 2 ml to achieve a final volume of 4 ml. Infra- diaphragmatic placement was confirmed by positive inspiratory pressure swings. The probe was then retracted until negative pressure swings during inspiration and maximal cardiac oscillations confirmed correct placement. ΔP_oes_ was calculated as the difference between the end-expiratory and end-inspiratory P_oes_.

### Ultrasonographic measurements

As respiratory muscle ultrasound is not routinely performed at our facility, the sonographer completed standardized training on the study protocol prior to data acquisition, and all ultrasound measurements were obtained by a single, trained investigator. Initial examinations were performed under supervision. Ultrasound data were acquired using a GE Vivid E9 ultrasound system (GE Healthcare, Chicago, IL, USA) equipped with a 9 L-D linear array transducer (frequency range 2.4–10.0 MHz). For diaphragm ultrasound, the transducer was placed in the right mid-axillary line to visualize the zone of apposition, where the diaphragm inserts into the rib cage^18^. In each phase, B-mode images were acquired to measure diaphragm thickness at end-inspiration (DT_insp_) and end-expiration (DT_exsp_). The diaphragm thickening fraction (DTF) was then calculated as a percentage according to Wait, Nahormek^19^using the following formula:

$$\:DTF=\frac{({DT}_{insp}-{DT}_{exsp})}{{DT}_{exsp}}\times\:100$$

For the intercostal ultrasound, the probe was placed vertically in the parasternal region between the second and third ribs, approximately 2–4 cm lateral to the sternum. The parasternal intercostal muscle appears as a three-layered biconcave structure. Two hyperechoic membranes outline the central muscle portion. Intercostal muscle thickness was measured at end-inspiration (IT_insp_) and end-expiration (IT_exsp_) in B-mode between the inner and outer hyperechoic fascial borders. ITF was calculated as^11^:

$$\:ITF=\frac{({IT}_{insp}-{IT}_{exsp})}{{IT}_{exsp}}\times\:100$$

Exemplary images of DTF and ITF measurements are provided as eFigure 1 and supplemental electronic material.

### Study phases

The study phases were randomized, ensuring that each participant underwent the same sequence within each set. The study consisted of three sets: seated at rest, ergometry with a value of 10 and 15 on the Borg RPE scale, respectively. Each set was further divided into five study phases, with the order of the phases randomised before the start of the experiment. During phase 0, participants were not connected to a ventilator. The ventilation settings during the different phases are summarised in Table 1. A Carescape™ R860 (GE HealthCare^®^, Chicago, USA) ventilator was used for ventilation and oesophageal pressure measurements. The order of the study phases within each set was randomised using a concealed allocation procedure with sealed, unmarked envelopes. The sonographer was blinded to NIV settings and exercise levels.

Measurements were taken after a uniform breathing pattern had been established, with a minimum waiting time of two minutes. This included DTF, ITF, ΔP_oes_, airway pressures, minute ventilation, blood pressure, heart rate, and peripheral oxygen saturation. The measurement and calculation of DTF, ITF, and ΔP_oes_ were based on two consecutive breaths.

**Table 1:** Phase settings.

	PEEP	ΔP_insp_
Phase I	No connection to the ventilator	–
Phase II	0 cmH_2_O	0 cmH_2_O
Phase III	5 cmH_2_O	0 cmH_2_O
Phase IV	5 cmH_2_O	5 cmH_2_O
Phase V	5 cmH_2_O	10 cmH_2_O

PEEP, positive end-expiratory pressure; ΔP_insp_, inspiratory pressure support.

### Statistical analysis

Repeated measures correlations between index tests were calculated using R software version 4.3.1 with the rmcorr-package^20^ version 0.5.4. All other statistical analyses were performed using IBM^®^ SPSS^®^ Statistics Version 29.0.1.0 (IBM, Armonk, US-NY). Due to the limited sample size, non-normally distributed data were assumed. Differences in measurements between exercise loads were compared using related-samples Friedman’s two-way analysis of variance by ranks (Friedman test). Summaries of categorical variables are given in terms of absolute numbers and group-related percentages. Summaries of metric variables are given as medians and interquartile ranges (IQR).

## Results

### Participants

43 subjects agreed to participate. In two subjects the nasogastric probe could not be placed. In three subjects, the study was prematurely terminated due to intolerance of the nasogastric probe. A total of 38 participants completed all measurements. A study flow chart is provided as eFigure 2.

Median age was 24.5 (IQR 22.0–28.0) years, median body mass index 25.3 (IQR 22.3–27.3) kg/m^2^ and 18 (52%) were female. At rest and during spontaneous breathing without connection to the ventilator the heart rate was 74 (IQR 71–86) bpm, DTF was 44.6 (IQR 15.6–78.2) % and ITF was 0.54 (IQR − 3.24–3.17) %. ΔP_oes_ was 7 (IQR 5–9) cmH_2_O. The baseline criteria of the 38 participants are shown in Table 2.

**Table 2:** Baseline characteristics. IQR, interquartile range; DTF, diaphragm thickening fraction; ITF, intercostal thickening fraction.

Number of participants, *n* (%)	38
Age [years], median (IQR)	25.3 (22.3–27. 3)
Female sex, n (%)	18 (52%)
Height [cm], median (IQR)	174.5 (168.0–180.0)
Weight [kg], median (IQR)	76.5 (68.0–88.3)
Body mass index [kg/m^2^], median (IQR)	25.3 (22.3–27.3)
DTF [%], median (IQR)	44.6 (15.6–78.2)
ITF [%], median (IQR)	0.54 (− 3.24–3.17)
Heart rate [bpm], median (IQR)	74 (71–86)

### Correlation between the parameters DTF, ITF and ΔP_oes_

Each participant completed three study sequences, each consisting of five randomised ventilation settings. One set of measurements was obtained for each combination of load and ventilatory support. Repeated measures correlation analysis revealed a moderate correlation between DTF and ΔP_oes_ (ρ = 0.419, *p* < 0.001). In contrast, no significant correlation was found between DTF and ITF (ρ = − 0.025, *p* = 0.572) or between ITF and ΔP_oes_ (ρ = − 0.040, *p* = 0.361). Repeated measures correlation plots are provided as eFigures 3–5.

### Discrimination between exercise loads

**Fig. 1:**
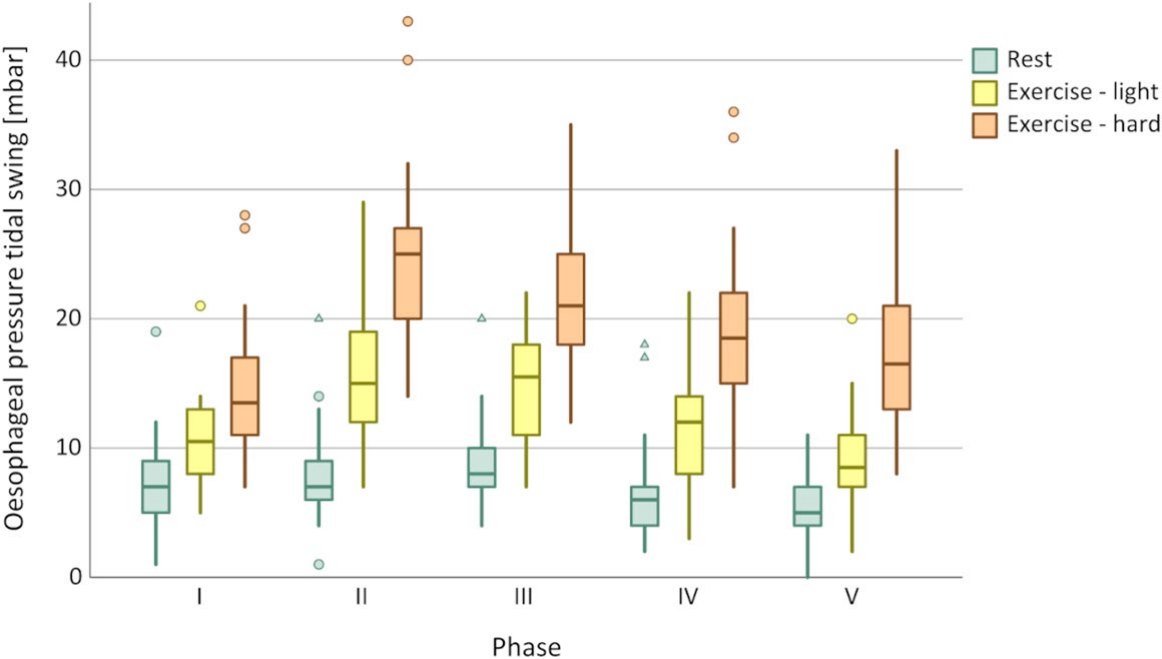
Discrimination of P_oes_ between exercise load according to respiratory phase. I = No ventilator; *p* < 0.001. II = EPAP 0 cmH_2_O/IPAP 0 cmH_2_O; *p* < 0.001. III = EPAP 5 cmH_2_O/IPAP 5 cmH_2_O; *p* < 0.001. IV = EPAP 5 cmH_2_O/IPAP 10 cmH_2_O; *p* < 0.001. V = EPAP 5 cmH_2_O/IPAP 15 cmH_2_O; *p* < 0.001. Circles indicate data outliers (third quartile + 1.5 interquartile range or first quartile – 1.5 interquartile range). Triangles indicate extreme outliers (third quartile + 3 interquartile range or first quartile – 3 interquartile range).

**Fig. 2:**
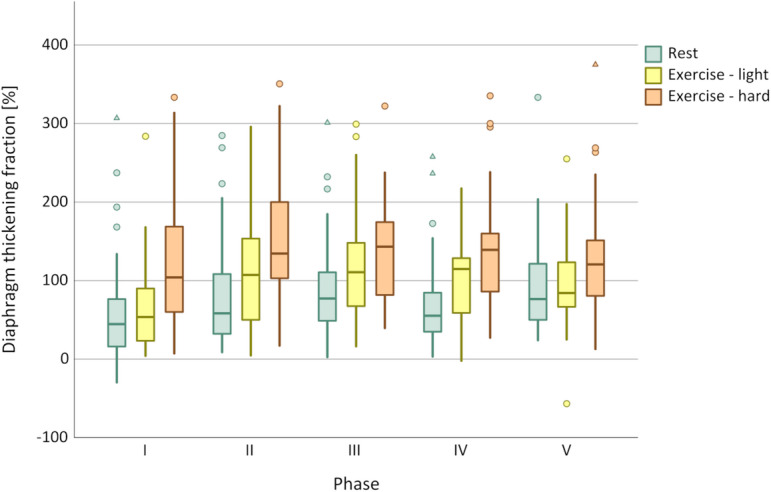
Discrimination of DTF between exercise load according to respiratory phase. I = No ventilator; *p* < 0.001. II = EPAP 0 cmH_2_O/IPAP 0 cmH_2_O; *p* < 0.001. III = EPAP 5 cmH_2_O/IPAP 5 cmH_2_O; *p* < 0.001. IV = EPAP 5 cmH_2_O/IPAP 10 cmH_2_O; *p* < 0.001. V = EPAP 5 cmH_2_O/IPAP 15 cmH_2_O; *p* < 0.002. Circles indicate data outliers (third quartile + 1.5 interquartile range or first quartile – 1.5 interquartile range). Triangles indicate extreme outliers (third quartile + 3 interquartile range or first quartile – 3 interquartile range).

**Fig. 3:**
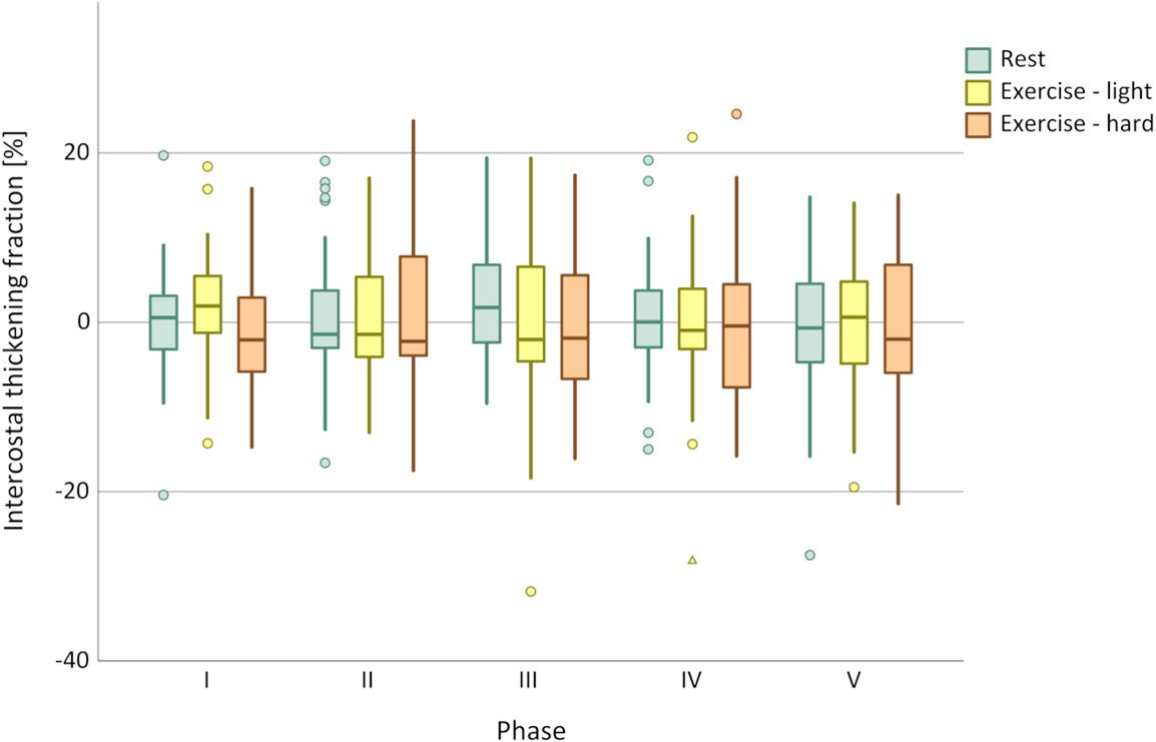
Discrimination of ITF between exercise load according to respiratory phase. I = No ventilator; *p* = 0.054. II = EPAP 0 cmH_2_O/IPAP 0 cmH_2_O; *p* = 0.855. III = EPAP 5 cmH_2_O/IPAP 5 cmH_2_O; *p* = 0.382. IV = EPAP 5 cmH_2_O/IPAP 10 cmH_2_O; *p* = 0.963. V = EPAP 5 cmH_2_O/IPAP 15 cmH_2_O; *p* = 0.913. Circles indicate data outliers (third quartile + 1.5 interquartile range or first quartile – 1.5 interquartile range). Triangles indicate extreme outliers (third quartile + 3 interquartile range or first quartile – 3 interquartile range).

In all ventilatory setups, both DTF and ΔP_oes_ increased with exercise load. While DTF permitted statistical discrimination between different exercise phases, ΔP_oes_ showed a clearer differentiation across all phases, as confirmed by the Friedman test and illustrated in Figs. 1 and 2. In contrast, ITF did not discriminate between the exercise loads (see Fig. 3). Table 3 summarises the median values of all respiratory parameters across the different study phases, together with the mean ranks used for the Friedman analysis.

**Table 3:** Median values and mean rank distribution across respiratory phases and exercise levels. Pinsp, inspiratory pressure support; RPE, rate of perceived exertion; DTF, diaphragm thickening fraction; ITF intercostal thickening fraction.

ΔPEEP	None			0			5			5			5		
ΔPinsp	None			0			0			5			10		
Borg RPE	0	10	15	0	10	15	0	10	15	0	10	15	0	10	15
ΔPoes [cmH_2_O]															
Median	7.0	10.5	13.5	7.0	15.0	25.0	8.0	15.5	21.0	6.0	12.0	18.5	5.0	8.5	16.5
Mean rank	1.2	2.0	2.8	1.1	2.0	2.9	1.1	2.0	2.9	1.2	2.0	2.8	1.1	1.9	3.0
*p*	< 0.001			< 0.001			< 0.001			< 0.001			< 0.001		
DTF [%]															
Median	45	54	104	58	107	134	77	111	143	55	115	139	77	84	121
Mean rank	1.7	1.7	2.6	1.5	2.0	2.6	1.4	2.2	2.4	1.4	2.0	2.6	1.6	2.0	2.4
*p*	< 0.001			< 0.001			< 0.001			< 0.001			0.002		
ITF [%]															
Median	0.5	1.9	− 2.1	− 1.4	− 1.4	− 2.2	1.7	− 2.0	− 1.9	0.0	1.0	− 0.4	− 0.7	0.6	− 2.0
Mean rank	2.0	2.2	1.9	2.0	2.0	2.0	2.0	2.1	1.9	2.0	2.0	2.0	2.0	2.0	2.0
*p*	0.054			0.855			0.382			0.963			0.913		

## Discussion

In this study, we demonstrated a moderate correlation between DTF and ΔP_oes_ at different ventilatory settings. These findings are consistent with the results of a previous study of our group, where both ΔP_oes_ and DTF showed similar behaviour^9^. Although associations between DTF and indices of respiratory effort have also been reported in critically ill patients, their strength varies across studies and clinical settings. Vivier et al. have described moderate to strong correlations under NIV after extubation^21^, whereas Menis et al. have not observed a clear relationship^22^. This could be attributable to a higher incidence of diaphragm dysfunction in invasively ventilated individuals, which may develop within a few days of mechanical ventilation^23,24^. In contrast, healthy individuals in our study showed no signs of diaphragm weakness. Evidence regarding diaphragm dysfunction in patients with de novo respiratory failure is limited, but preserved diaphragm function is likely more common in early disease stages, allowing sonographic evaluation of respiratory effort during NIV^25^.

No correlation was observed between ΔP_oes_ and ITF or between ITF and DTF. Furthermore, ITF did not discriminate between different levels of respiratory effort in our study, suggesting that ITF was not a reliable marker under the given conditions. These findings are in line with previous observations in spontaneously breathing healthy individuals where no significant change in intercostal muscle thickness during different respiratory manoeuvres was reported^15^. This could be due to the predominantly isometric contraction of the intercostal muscles, which stabilise the thoracic cage and prevent paradoxical movements without significant muscle shortening or thickening^15^despite the presence of inspiratory electrical activity in the parasternal intercostal muscles^26^. Additionally, the limited usefulness of ITF in our study may in part reflect the fact that parasternal intercostals do not necessarily represent the workload of the entire group of accessory inspiratory muscles in healthy subjects^27^. While this may be true for resting or moderate breathing, Yoshida et al. demonstrated that the thickness of the parasternal intercostal muscles can reflect maximal breathing efforts compared to normal breathing in healthy men^14^. As differentiation between these states can usually be attained by clinical inspection alone, such extreme respiratory efforts were not intended and not performed by any of the participants in the present study.

ITF could be more informative in patients with diaphragmatic dysfunction^12,13^. Dres et al. suggested that parasternal intercostal ultrasound may help to assess the imbalance between respiratory effort and respiratory capacity in mechanically ventilated patients^12^. Umbrello et al. found that combining diaphragm and intercostal thickening measurements in mechanically ventilated patients could help to differentiate between true low respiratory effort and high effort in the presence of diaphragmatic dysfunction^13^. As those individuals should rely on the accessory respiratory muscles more than others^28^, a more significant recruitment of the intercostal muscles could be observable.

Taken together, these findings suggest that in healthy individuals, increased respiratory demands can still be compensated for by the diaphragm alone, whereas in the presence of diaphragm dysfunction, the intercostal muscles may play a greater role.

### Limitations of this study

This study was conducted in healthy young volunteers. The sample size was relatively small, which may have limited the statistical power to detect smaller effects. Finally, the measurements were made under standardised conditions and without long-term follow-up, so the results may not reflect the variability and complexity of clinical scenarios over time. No direct conclusions for clinical practice or patient management should be drawn from these experimental findings.

## Conclusions

While DTF showed a moderate correlation with ΔP_oes_ and allowed reasonable discrimination between exercise loads in noninvasively ventilated healthy subjects, ITF did not correlate with ΔP_oes_ or DTF and did not discriminate between exercise levels. DTF may reflect relative changes in respiratory effort and may support individual titration of noninvasive respiratory support. In healthy individuals, ITF was not informative. Further studies are needed to clarify the clinical utility of ITF in diseased noninvasively ventilated patients.

## Supplementary Information

Below is the link to the electronic supplementary material.

Supplementary Material 1

Supplementary Material 2

Supplementary Material 3

Supplementary Material 4

Supplementary Material 5

Supplementary Material 6

## Data Availability

The data that support the findings of this study are available from the corresponding author upon reasonable request.

## Abbreviations

ΔP_insp_: Inspiratory pressure support
ΔP_oes_: Tidal oesophageal pressure swings
DTF: Diaphragm thickening fraction
ITF: Intercostal thickening fraction
