# Supplementary material for: Intercostal thickening fraction adds no value to diaphragm thickening fraction in healthy subjects undergoing noninvasive ventilation
Source: Sci Rep. 2026 Feb 17;16:7165. doi: 10.1038/s41598-026-40192-4 (PMC12920780; doi:10.1038/s41598-026-40192-4)
Supplement: Supplementary file 1 — Supplementary Material 1 [file 41598_2026_40192_MOESM1_ESM.docx]

**SUPPLEMENTAL MATERIAL**

Supplement to:

**Intercostal Thickening Fraction Adds No Value to Diaphragm Thickening Fraction in Healthy Subjects Undergoing Noninvasive Ventilation**

Clara Hoermann et al.

**Table of Contents**

Supplemental Figure 1 – Measurement examples ………………………………………………2

Supplemental Figure 2 – Study flow chart …………………………………………….…………..3

Supplemental Figures 3-5 – Repeated measures correlation plots …….....…………………4-5

Supplemental Videos 1-4 Ultrasonographic diaphragm and intercostal loops…………………5

**Supplemental Figure 1 – Measurement examples for diaphragm and intercostal thickness**


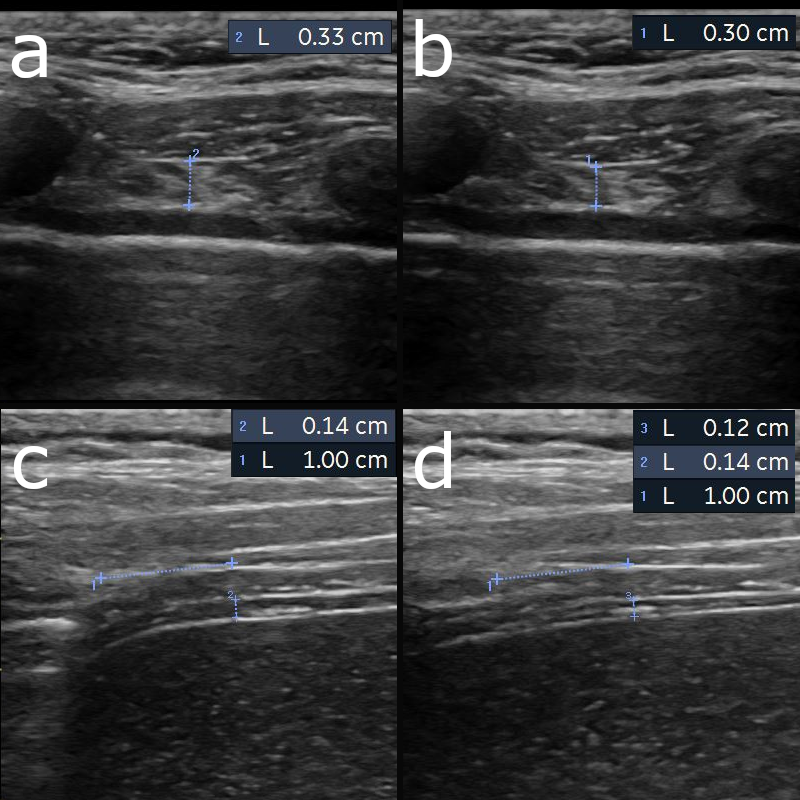


a) End-inspiratory intercostal thickness. b) End-expiratory intercostal thickness. c) End-inspiratory diaphragm thickness, measured 1 cm from the pleural recess. d) End-expiratory diaphragm thickness with the same distance marking to the pleural recess to maintain the same location for measurements

**Supplemental Figure 2 – Study flow chart**

**
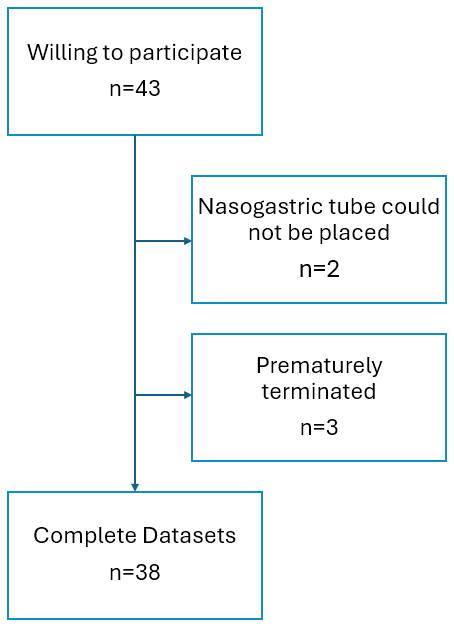
**

**Supplemental Figure 3 – Repeated measures correlation plot for oesophageal pressure swings and diaphragm thickening fraction**


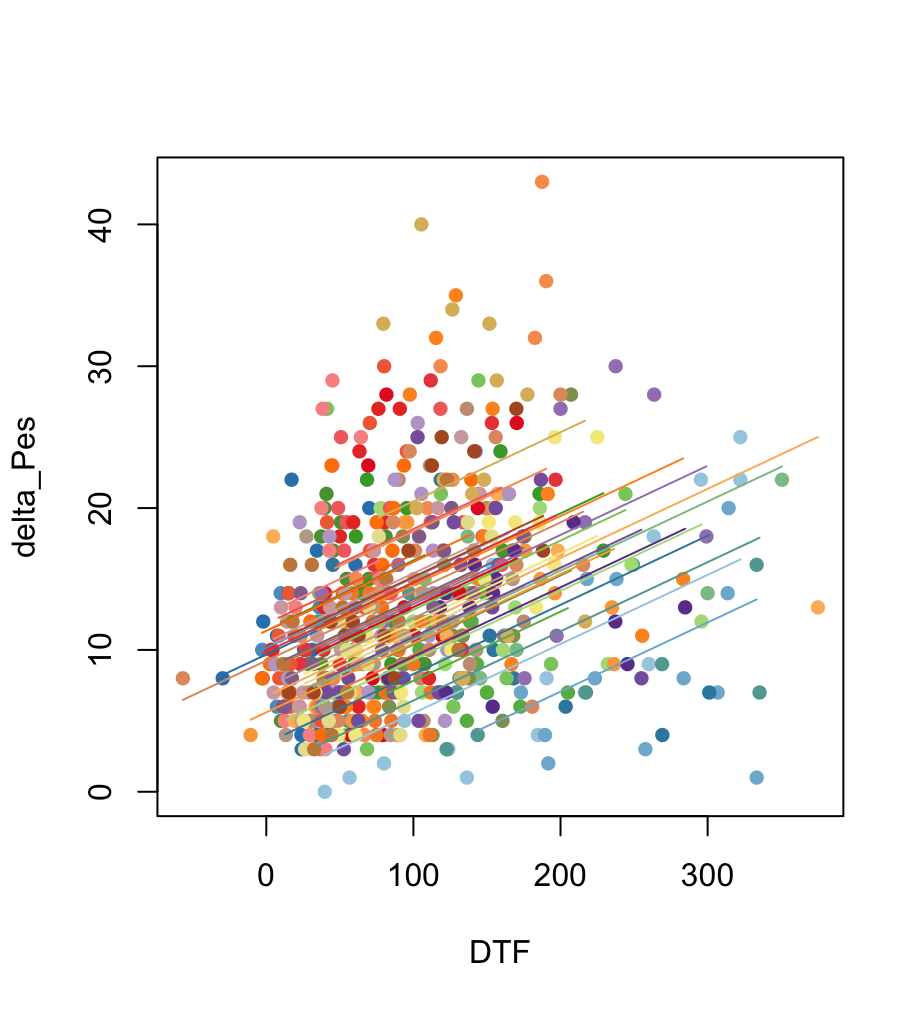


**Supplemental Figure 4 – Repeated measures correlation plot for intercostal thickening fraction and diaphragm thickening fraction**


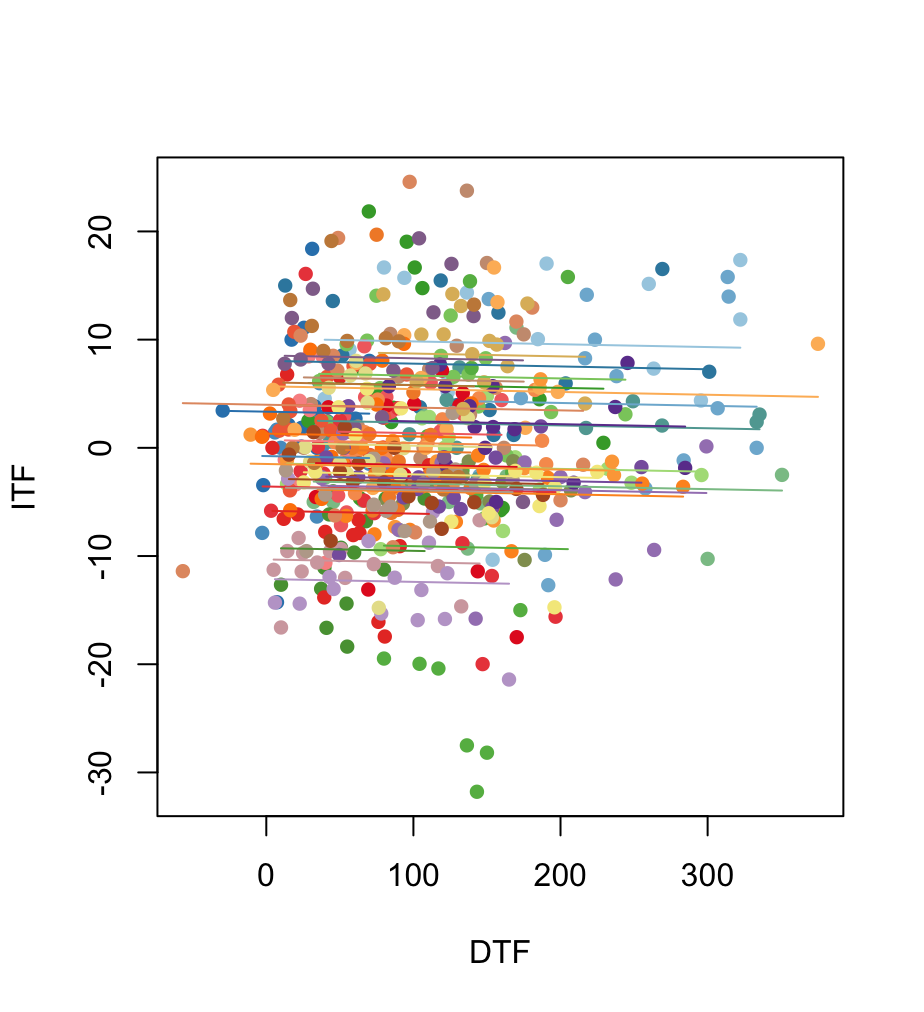


**Supplemental Figure 5 – Repeated measures correlation plot for intercostal thickening fraction and oesophageal pressure swings**


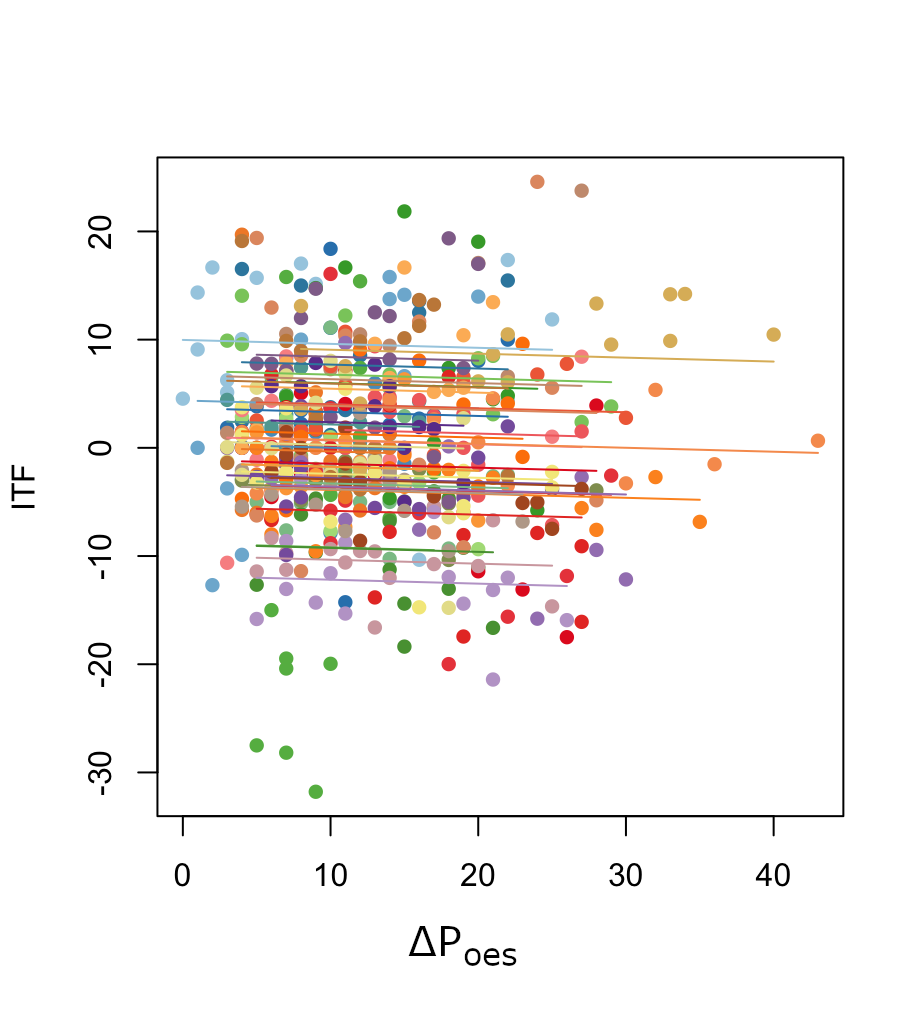


**Supplemental Videos 1-4**

1 Diaphragm loop during rest

2 Diaphragm loop during exercise

3 Intercostal loop during rest

4 Intercostal loop during exercise
