# Supplementary material for: Intercostal thickening fraction adds no value to diaphragm thickening fraction in healthy subjects undergoing noninvasive ventilation
Source: Sci Rep. 2026 Feb 17;16:7165. doi: 10.1038/s41598-026-40192-4 (PMC12920780; doi:10.1038/s41598-026-40192-4)
Supplement: Supplementary file 6 — Supplementary Material 6 [file 41598_2026_40192_MOESM6_ESM.pdf]

Proportion of Diaphragm and Intercostal Thickening Fractions During Noninvasive Ventilation in Healthy Subjects

Study design:	Physiological study
Short Title	DTF-ITF-NIV
Sponsor:	none
Protocol Date:	27.03.2024

Study Principal Investigator

Simone Britsch MD

Head of the Medical Intensive Care Unit 10-4/11-4
I. Department of Medicine
Cardiology, Angiology, Pneumology
Medical Intensive Care Unit, Hemostaseology
Theodor-Kutzer-Ufer 1-3

Study Investigators:

Simon Lindner MD

Burcu Link MS

Luisa Drotleff MS

Lena Doerflinger MS

Medical Intensive Care Unit 10-4/11-4
I. Department of Medicine
Cardiology, Angiology, Pneumology
Medical Intensive Care Unit, Hemostaseology

Theodor-Kutzer-Ufer 1-3

ABSTRACT

Context:

Non-invasive ventilation (NIV) can be used in acute respiratory failure to delay and possibly even prevent invasive mechanical ventilation. Through support pressure (Pinsp), NIV can reduce the patient's respiratory effort. Estimation of respiratory effort may be useful to titrate Pinsp to individual requirements. Previous experiments have indicated usefulness of the diaphragm thickening fraction (DTF) for this purpose. Other study groups have proposed that additional assessment of the intercostal muscle thickening fraction (ITF) may further improve this estimation, however this has never been tested during noninvasive ventilation.

Objectives:

This study aims to test proportions and correlation of DTF and ITF in different respiratory effort conditions induced by exercise load during NIV of healthy volunteers.

Study Design:

This is a physiological study.

Setting/Participants:

The study is performed in the I. Medical department.

Healthy volunteers will be recruited for this study.

1 BACKGROUND INFORMATION AND RATIONALE

Non-invasive ventilation (NIV) can be used in acute respiratory failure to avoid invasive mechanical ventilation. By providing supportive pressure, NIV can reduce the patient's respiratory effort. Estimating the respiratory effort can be helpful in adapting ventilation to individual requirements. Studies have shown that inspiratory diaphragmatic thickness (DTF) increase is useful for this purpose. Other study groups have suggested that an additional measurement of intercostal muscles (ITF) could further improve this estimate, but this has never been tested during non-invasive ventilation. The aim of this study is to investigate the respective proportion and correlation of DTF and ITF under different conditions of respiratory effort elicited by exercise during non-invasive ventilation in healthy volunteers.

1.1 Compliance Statement

All investigators will ensure that this trial is conducted in accordance with the principles of the Declaration of Helsinki, relevant regulations, and Good Clinical Practice. The investigators will perform the study in accordance with this protocol. Collection, recording, and reporting of data will be accurate and will ensure the privacy, health, and welfare of research subjects during and after the study.

2 STUDY OBJECTIVES

2.1 Primary Objective

The purpose of the study is to assess whether ITF adds relevant information on respiratory effort measurements during NIV compared to DTF. As a comparator, respiratory effort will be measured using oesophageal pressure swings.

2.2 Secondary Objectives

The secondary objectives are to determine the correlation of DTF, ITF and oesophageal pressure swings and the influence of increased respiratory effort induced by exercise on their relationships. Another secondary objective is to assess the effect of different levels of respiratory support (spontaneous breathing, CPAP, pressure support) on DTF, ITF and oesophageal pressure swings. Furthermore, factors influencing the correlation of DTF, ITF and oesophageal pressure swings should be identified.

3 INVESTIGATIONAL PLAN

3.1 General Schema of Study Design

This is a single centre physiological study of healthy subjects receiving non-invasive respiratory support.

3.2 Study Duration and Enrolment

Recruitment will be started when the ethics committee has approved the study protocol and all additional documents i.e. consent form. Over the course of approximately four months all subjects taking part in this project will be enrolled.

3.3 Total Number of Subjects Projected

The aimed number of included healthy subjects is 50.

3.3.1 Duration of Study Participation

Each subject's participation will include ultrasonographic measurement of DTF, ITF and oesophageal manometry during a set of NIV modes. Each participant's participation will last 30 Minutes. The entire study is expected to last about four months. A prolonged recruitment period will be discussed when too few participants have been enrolled after the projected time interval.

3.4 Study Population

3.4.1 Inclusion Criteria

1. Volunteers aged 18 or older
2. Ability to give written informed consent

3.4.2 Exclusion Criteria

1. Known bleeding disorder, history of recurrent bleeding or current anticoagulant therapy
2. Allergy to ultrasound gel or external agents containing plastic (NIV mask, gastric tube)
3. Refusal of or medical contraindication to non-invasive ventilation

4 STUDY PROCEDURES

4.1 Screening Visit

At the screening visit, the following points will be evaluated:

Medical History

Current Medication

Informed Consent

Inclusion criteria

Exclusion criteria

4.2 Measurement Period

SetupSubjects take part in the study once. Participants will be seated on a semi-recumbent bicycle ergometer during the whole experiment. At the beginning, participants will be asked to determine their ergometry exercise loads to achieve perceived exertion equivalents to Borg scale 10 (fairly light) and 15 (hard). Participants will undergo three sets of 5 study phases each. The order of the study phases will be randomly assigned, but with the same order of phases in each set.

- First set: seated on the ergometer, but resting
- Second set: ergometry with perceived exertion Borg scale 10
- Third set: ergometry with perceived exertion Borg scale 15

As mentioned above, each set is subdivided into an identical order of 5 study phases. The order of phases is randomly assigned prior to the start of the experiment. The 5 different phases are:

- Without connection to a respirator (phase null)
- Connected to a respirator with PEEP and Psupp of 0 cmH_2O (phase P0S0)
- Connected to a respirator with PEEP of 5 cmH_2O and Psupp of 0 cmH_2O (phase P5S0)
- Connected to a respirator with PEEP of 5 cmH_2O and Psupp of 5 cmH_2O (phase P5S5)
- Connected to a respirator with PEEP of 5 cmH_2O and Psupp of 10 cmH_2O (phase P5S10)

The DTF, ITF, airway and oesophageal pressures as well as minute volume, blood pressure, heart rate and SpO2 will be recorded at the end of each study phase.

4.3 Follow-up

There is no follow up for this study.

4.4 Subject Completion/Withdrawal

Subjects may withdraw from the study at any time. They may also be discontinued from the study at the discretion of the investigator for lack of adherence to study measurements. The

Investigator may exclude participants from this study to protect the subject for reasons of safety or for administrative reasons. It will be documented whether or not each subject completes the clinical study. If the Investigator becomes aware of any serious, related adverse events after the subject completes or withdraws from the study, they will be recorded in the source documents and on the CRF.

5 STATISTICAL CONSIDERATIONS

5.1 Primary Objectives

The purpose of the study is to assess whether ITF adds relevant information on respiratory effort measurements during NIV compared to DTF. As a comparator, respiratory effort will be measured using oesophageal pressure swings.

5.2 Secondary Endpoints

The secondary objectives are to determine the influence of increased respiratory effort induced by exercise on absolute values of the index tests and their relationships. Another secondary objective is to assess the effect of different levels of respiratory support (spontaneous breathing, CPAP, pressure support) on DTF, ITF and oesophageal pressure swings.

5.3 Statistical Methods

5.3.1 Primary Objective

Oesophageal pressure swings are used as the comparator for respiratory effort. Correlations with DTF and ITF will be assessed by calculating repeated measures correlation coefficient. Correlations of DTF and ITF will be additionally visualized using clustered box plots.

5.3.2 Secondary Objectives

Differences between measurements in the three sets and/or five phases will be compared using the related-samples Friedman's two-way analysis of variance by ranks and visualized by simple box plots according to set/phase.

Subgroup analyses by gender, BMI, and exploratory analysis of factors influencing the correlation of DTF, ITF and the oesophageal pressure fluctuation.

5.4 Sample Size and Power

In a previous pilot experiment with 23 participants, we studied the correlation of DTF and oesophageal pressure swings, where we found a moderate correlation and fair discrimination between exercise loads of DTF and oesophageal pressure swings (not yet published). Furthermore, we observed inter-individual differences in the DTF/oesophageal pressure relationship, that might be explained by habitus, BMI or gender. However, the small sample size prevented any meaningful evaluation of this question. No other comparable studies exist for this study question. Formal sample size determination cannot be calculated, the authors conjecture that 50 participants will be needed to adequately control for confounding factors.

5.5 Interim Analysis

No interim analysis is planned.

6 SAFETY MANAGEMENT

6.1 Clinical Adverse Events

Clinical adverse events (AEs) will be monitored throughout the study.

6.2 Adverse Event Reporting

Since the study procedures are not greater than minimal risk, AEs are not expected. If any unanticipated problems related to the research involving risks to subjects or others happen during this study (including AEs) these will be reported to the chief investigator. Through discussion of the study team, it will be decided whether any AE may be linked to any of the study measures, and whether exclusion of the participant or discontinuation of the study must be undertaken.

7 STUDY ADMINISTRATION

7.1 Data Collection and Management

Source documents are where data are first recorded, and from which participants' CRF data are obtained. All documents will be stored safely in confidential conditions. On all trial-specific documents, other than the signed consent, the participant will be referred to by the trial participant number/code, not by name.

Information will be collected by a study investigator for each participant using a data collection form. The form will be stored in the clinical report file (CRF). The participants will be identified by a unique trial specific number in any database. The name and any other identifying detail will NOT be included in any trial data electronic file.

No data, forms, or other records will leave the site to maintain confidentiality. All records will be kept in a locked file cabinet. All computer entry and networking programs are secured with personalized passwords. Data will be retained for 10 years on the institutions premises and will afterwards be deleted.

Participants are given the option to be approached for future research, this is contained in the consent form. Those contact details are held securely, separately from the research data, and kept updated.

7.2 Regulatory and Ethical Considerations

7.2.1 Risk Assessment

None of the tests hold relevant risks for participants. Patient safety will, however, be routinely assessed in the clinical report file of each patient. Screening for any AE will be part of any study visit.

7.2.2 Potential Benefits of Study Participation

No individual benefit is estimated for the subjects.

7.2.3 Risk-Benefit Assessment

As there are minimal risks associated with this study, the low potential of benefit for participants is justifiable. Furthermore, future patients might benefit from findings of this study.

7.3 Recruitment Strategy

All persons that fulfil the inclusion criteria are eligible for this study.

7.4 Informed Consent/Assent and HIPAA Authorization

Written consent for screening and participation in the trial is required.

Before any screening procedure is performed, informed consent must be obtained. Both the screening and study procedures will be summarized in a single informed consent form.

The participant must personally sign and date the latest approved version of the informed consent form before any trial specific procedures are performed.

Written and verbal versions of the participant information and informed consent will be presented to the participants detailing no less than: the exact nature of the trial; what it will involve for the participant; the implications and constraints of the protocol; any risks involved in taking part. It will be clearly stated that the participant is free to withdraw from the trial at any time for any reason without prejudice to future care, without affecting their legal rights and with no obligation to give the reason for withdrawal.

The participant will be allowed as much time as wished to consider the information, and the opportunity to question the Investigator or other independent parties to decide whether they will participate in the trial. Written Informed Consent will then be obtained by means of participant dated signature and dated signature of the person who presented and obtained the Informed Consent. The person who obtained the consent must be suitably qualified, experienced and have been authorized to do so by the Chief/Principal Investigator. A copy of the signed Informed Consent will be given to the participant. The original signed form will be retained at the trial site in the case report folder of the respective centre. Additional digital documentation of the consent will be made in a central study registry.

8 PUBLICATION

Any presentation, abstract, or manuscript of trial data will be made available for review by all participating study investigators.

9 REFERENCES

1. Goligher EC, Fan E, Herridge MS, et al. Evolution of Diaphragm Thickness during Mechanical Ventilation. Impact of Inspiratory Effort. Am J Respir Crit Care Med. 2015;192(9):1080-1088. doi:10.1164/rccm.201503-0620OC

2. Jansen D, Jonkman AH, Vries HJ, et al. Positive end-expiratory pressure affects geometry and function of the human diaphragm. J Appl Physiol (1985). 2021;131(4):1328-1339. doi:10.1152/japplphysiol.00184.2021

3. Francis CA, Hoffer JA, Reynolds S. Ultrasonographic Evaluation of Diaphragm Thickness During Mechanical Ventilation in Intensive Care Patients. Am J Crit Care. 2016;25(1):e1-e8. doi:10.4037/ajcc2016563

4. Lassola S, Miori S, Sanna A, Cucino A, Magnoni S, Umbrello M. Central venous pressure swing outperforms diaphragm ultrasound as a measure of inspiratory effort during pressure support ventilation in COVID-19 patients. J Clin Monit Comput. 2022;36(2):461-471. doi:10.1007/s10877-021-00674-4
